# The Research Status and Prospects of *Floccularia luteovirens*: A Mycorrhizal Fungus with Edible Fruiting Bodies

**DOI:** 10.3390/jof9111071

**Published:** 2023-11-01

**Authors:** Yanqing Ni, Luping Cao, Wensheng Li, Qin Zhang, Rencai Feng, Zhiqiang Zhao, Xu Zhao

**Affiliations:** 1 Institute of Urban Agriculture, Chinese Academy of Agricultural Sciences, Chengdu 610299, China; niyanqing0214@163.com (Y.N.); jeanzhang666@yeah.net (Q.Z.); fengrencai@caas.cn (R.F.); 2 College of Food and Biological Engineering, Chengdu University, Chengdu 610106, China; li121498@163.com; 3 College of Life Science and Technology, Gansu Agricultural University, Lanzhou 730070, China; caolup@163.com; 4 Chengdu National Agricultural Science and Technology Center, Chengdu 610299, China; 5 Zhuoni County Agricultural Technology Extension Station, Gannan 747600, China; znzhiqiang@163.com

**Keywords:** *Floccularia luteovirens*, ecological characteristics, biological characteristics, domestication and cultivation, food and medicinal value

## Abstract

*Floccularia luteovirens*, a rare wild edible and medicinal fungus, is endemic to the Tibetan plateau. However, attempts to artificially domesticate this species have not been successful, resulting in extremely limited utilization of this valuable resource. This paper presents the geographical distribution of *F. luteovirens,* along with its ecological and biological characteristics. It explores population relations, symbiotic relationships, soil microbial community relations, fruiting body occurrence conditions, nutritional metabolism, and reproductive patterns. The cultivation techniques, as well as the edible and medicinal value of this mushroom, are also reviewed. Through an overall analysis of the physiological characteristics and current research status of *F. luteovirens*, the paper discusses its development prospects. The aim is to provide a reference for other researchers and promote its artificial domestication, resource development, and utilization.

## 1. Introduction

Edible fungi are a diverse group of large fungi with unique physiological characteristics and a wide range of forms. They are known for their delicious taste, rich nutrition, and diverse bioactive ingredients, making them a popular choice for healthy eating [1]. Among edible fungi, mycorrhizal fungi are a special type that form a symbiotic relationship with plants, relying on host plants to provide the necessary nutrients for their growth [2]. Research has shown that many mycorrhizal edible fungi have higher protein and trace element contents compared to saprophytic edible fungi [3,4,5]. *Floccularia luteovirens* (*Alb. & Schwein.*) *Pouzar* is a rare wild edible fungus found in the Qinghai–Tibet Plateau of China. It forms mycorrhizal symbiosis with *Kokanica humilis* and plays an important role in the alpine meadow or grassland ecosystem [6,7]. This mushroom is a local delicacy and has significant economic value. Additionally, it has been used as a traditional Tibetan medicine with notable effects in hypoglycemic, antioxidant, anti-tumor, and immune regulation [8]. Therefore, *F. luteovirens* holds great potential as a resource for functional food or medicine. However, as a local resource, the research on *F. luteovirens* is still very limited. Many aspects of this species remain unknown, such as the specific mechanism of fruiting body production, artificial culture conditions for mycelium, and the form of action of pharmacologically active ingredients. The inability to achieve commercial cultivation further restricts its processing and utilization. Additionally, environmental changes and overpicking contribute to the increasing scarcity of this resource. This review aims to synthesize the existing fragmented information about *F. luteovirens* and comprehensively analyze its ecological and biological characteristics, as well as its food and medicinal value, so as to raise awareness among researchers about this species and encourage further study on its conservation, domestication, and utilization.

## 2. Taxonomy of *F. luteovirens*

**Figure 1:**
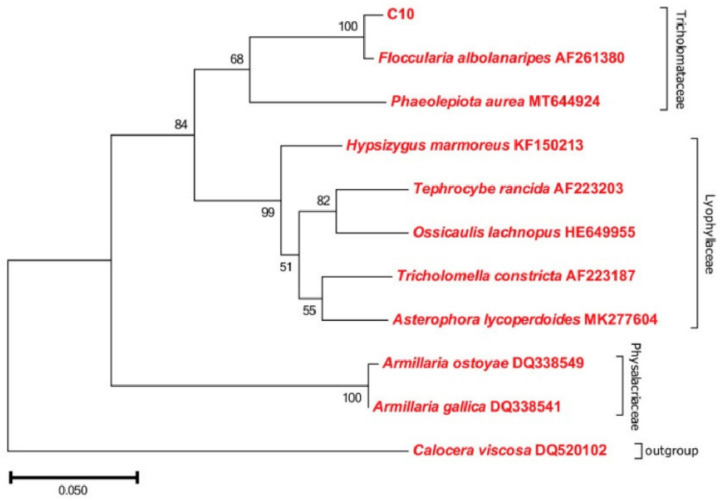
Phylogenetic analysis of *F. luteovirens* C10 based on the ITS gene sequence. (LIU, 2021 [13]).

*F. luteovirens* is a member of the Basidiomycota phylum, belonging to the Agaricomycetes class, the Agaricales order, and the Agaricaceae family within the Floccularia genus [9]. This edible mushroom is commonly referred to as a golden mushroom or grassland yellow mushroom by local residents [10]. The taxonomic status of *F. luteovirens* has been controversial and misunderstood for a long time. It was previously thought to belong to the genus Armillaria, under the Physalacriaceae family, and was therefore referred to as *Armillaria luteovirens* in various studies [11], and is still used today. Gan et al. [12] assembled and annotated the genome of *F. luteovirens*. Through phylogenetic tree analysis, they determined that *F. luteovirens* belongs to the Agaricaceae family and that its species differentiation occurred approximately 170 million years ago. Liu et al. [13] conducted rDNA-ITS sequence determination on 36 *F*. *luteovirens* strains isolated from field collection in Qinghai Province, and then, combined with comparative genomic data, further confirmed that *F. luteovirens* should be classified under the genus Floccularia rather than the genus Armilaria. The NJ phylogenetic tree showed that the *F. luteovirens* C10 strain was closer to the species of Tricholomelaceae in a phylogenetic relationship and clustered with *Floccularia albolanaripes* with a bootstrap value of 99% (Figure 1). As a result, the NCBI database has also replaced the species *Armillaria luteovirens* with *Floccularia luteovirens* (NCBI: txid493452) in 2019. 

## 3. Ecological Characteristics of *F. luteovirens*

### 3.1. Geographical Distribution and Population Relationship of F. luteovirens

*F. luteovirens* is primarily found on the Qinghai–Tibet Plateau in China. Its geographical distribution range in China is approximately latitude 28°93′–37°69′ N and longitude 90°4′–102°1′ E [7]. It is most commonly found in the grasslands or alpine meadows of Qinghai and Tibet, with smaller populations in the western part of Sichuan and the southern part of Gansu [14]. This species has also been reported in the Altai Republic of Russia and Mexico [15,16]. According to reports [6,17,18], the altitude distribution range in Qinghai province is around 3000 to 4300 m, with the majority of sightings occurring in meadows between 3200 and 3800 m above sea level. The main areas of production include Haibei, Huangnan, Hainan, Guoluo, Yushu, and other regions. In Tibet, the altitude distribution ranges from about 3200 to 4600 m. The highest point in the Qomolangma region can reach up to 5000 m above sea level. Significant production areas for *F. luteovirens* include Lhasa, Dazi, Langkazi, Pali, Bangda, Dingri, and others. In Shiqu County, Sichuan Province, *F. luteovirens* mainly grows in the grasslands along the Yalong River at an altitude of 4000–4300 m. The geographical structure of *F. luteovirens* exhibits significant differences. A comprehensive analysis of 91 samples from 10 geographical groups in Qinghai, Tibet, and Sichuan provinces revealed that the population genotype comprises two branches. This study also found that population genetic variation primarily originates from within the population, with substantial genetic differentiation observed between populations [19]. Moreover, the genotype diversity (h) of *F. luteovirens* ranged from 0.385 to 0.876, indicating a moderate level of genetic diversity [20].

### 3.2. Symbiotic Relationship of F. luteovirens

*F. luteovirens* is a symbiotic mycorrhizal fungus that forms a partnership with the Kobresia plants. The hyphae of this fungus are distributed in the soil at a depth of 5–30 cm. During the fruiting bodiy harvest season, white mycelia can be observed on the roots of *Kobresia humilis* [6]. Although many scholars agree that *F. luteovirens* is an ectomycorrhizal fungus [20,21], there is a lack of anatomical or modern biological identification, and its mycorrhizal types need to be further clarified. Ectomycorrhiza symbiosis is a widespread association between the roots of woody plants and soil fungi in forest ecosystems [22]. Examples of mycorrhizal edible fungi include matsutake, boletus edulis, and truffle, which form symbiotic relationships with pine or oak trees [23]. However, the fruiting body of *F. luteovirens* occurs in alpine meadows, where the dominant vegetation consists of *K*. *humilis* and alpine weeds [24]. In Guo et al.’s study, they used a metagenomic method to determine the diversity of endophytic fungi in *K*. *humilis*. They found that *F. luteovirens* is one of the dominant endophytic fungi in *K*. *humilis* [25]. The habitat of *F. luteovirens* often forms a ribbon-like fairy ring, with a circle width of approximately 50 cm and a diameter of about 6 m [24]. According to Shantz and Piemeisel’s classification of fairy rings, the fairy ring formed by *F. luteovirens* falls under type II [26]. This means that the existence of the fairy rings stimulates the growth of plants, and there is only a green grass ring without a dry grass ring. Fairy rings are a well-known ecological landscape found in grasslands, and a similar phenomenon can be observed in the fairy ring formed by *Leucocalocybe mongolica* in the Mongolian Plateau [27]. Additionally, the plants within the *F. luteovirens* ring exhibit higher biomass, plant height, and plant density compared to those outside the ring [6,28]. The formation of a fairy ring is a distinctive occurrence resulting from the growth of fungal mycelium in the soil. Several studies have demonstrated [29,30,31] that Fairy Ring fungi can produce plant growth regulatory substances to stimulate plant growth, such as 2-azahypoxanthine (AHX) and imidazole-4-carboxamide (ICA), and regulate the activity of various enzymes in the soil, thereby improving soil characteristics and providing favorable conditions for plant growth. The plants on the fairy rings displayed a noticeable increase in greenness, and the abundance index and species diversity index of the plant communities on the rings were significantly higher. The promotion of plant growth by *F. luteovirens* is closely linked to the mycorrhizal system. On the one hand, plants are able to contribute to the decomposition and activation of mineral elements in the soil through a vast network of hyphae, particularly enhancing the absorption of organophosphorus and other nutrient elements. On the other hand, plants can also serve as a source of carbon for fungi in the form of sugar or fatty acids [32]. *F. luteovirens* can change minerals into usable organic forms by releasing organic acids. This makes roots absorb more minerals and improves the stress resistance of symbiotic plants. [33]. Physiological and metabolomic analysis has shown [34] that *F. luteovirens* can influence the accumulation of soil metabolites, regulate plant carbon/nitrogen metabolism, and enhance the growth of above-ground tissues in alpine meadow plants. Meanwhile, it may also reduce root growth to adjust the root-shoot ratio, consequently increasing nutrient accumulation in plants. In addition, the mycelium of *F. luteovirens* also produces volatile organic compounds (VOCs) that regulate the distribution of root auxin, thereby controlling the root development of plants. This, in turn, affects the growth, metabolism, and environmental adaptability of plants [35]. Andrea Polle et al. have also discovered that ECM fungi produce volatiles, particularly terpenoid derivatives, which can influence the development of host plants and their lateral roots [36]. This finding is similar to the role of *F. luteovirens* in regulating plant growth. The fairy ring effect, caused by the growth and metabolism of mycelia, has significant impacts on the plant-soil ecosystem in the habitat. It can alter the inter-specific competitive relationship of grassland communities and influence the composition and direction of plant community succession [37]. However, the growth of *F. luteovirens* has a minimal effect on the uniformity and succession of plant communities [24]. In conclusion, as an endemic species, *F. luteovirens* plays a positive role in regulating plant growth and maintaining ecosystem stability in the alpine meadows of the Qinghai–Tibet Plateau.

### 3.3. Soil and Soil Microbial Communities

The soil of the *F. luteovirens* habitat is dark, with a pH range of 6.8–7.2 and a humus layer measuring 10–20 cm. Below the humus of the soil is the parent material, containing gravel and grass roots [6,18]. The decomposition of fungal mycelia can enhance the mineralization of soil organic matter and release nutrients, thereby enriching the soil and significantly increasing the availability of nutrients. In the fairy ring area, the soil within the ring exhibits higher levels of nutrients and enzyme activities compared to both the inside and outside areas of the ring [38]. In the 0–10 cm soil layer, the soil water content, available phosphorus, nitrate nitrogen, and ammonia nitrogen content in the fairy ring formed by *F. luteovirens* were significantly higher than outside the circle [24,33]. Floccularia is the absolute dominant genus among soil microorganisms in *F. luteovirens* nest soil, with a relative abundance of 85.76%. The FunGuild function predicted that the soil fungi in the habitat of *F. luteovirens* were mainly symbiotic, followed by saprophytic [39]. This finding aligns with the mycorrhizal symbiotic nature of *F. luteovirens*. The presence of *F. luteovirens* in the soil has a regulatory effect on the microbial community. In the area where mycelia grow, the diversity of bacteria increases while the diversity of fungi decreases [33,40]. The possible reason is that the interspecific cooperation in mycorrhizal symbiosis between *F. luteovirens* and *K. humilis* enhances the competitive ability of *F. luteovirens* and inhibits the vitality of other fungal species in a limited ecological niche. Ectomycorrhizal (ECM) fungi play a crucial role in the rhizosphere community by interacting with various microorganisms. One of the main interactions involves the competition between different ECM fungi as they strive to occupy the root space of the host in order to obtain additional carbon sources [41]. A study on the fairy ring formed by *F. luteovirens* revealed interesting findings. The number of operational taxonomic units (OTUs) in the three regions was as follows: 300 in the IN region, 1107 in the ON region, and 14 in the OUT region. No other ECM fungi were detected in any of the three regions, with only a small amount of arbuscular mycorrhizal fungi being detected. These results suggest potential competition between *F. luteovirens* and other ECM species [33]. The presence of *F. luteovirens* in the soil has indirect effects on the composition and metabolism of soil microorganisms. This, in turn, leads to changes in the distribution and composition of metabolites in the soil. It is important to note that microorganisms in the soil have an impact on the development of mycelia, mycorrhizal synthesis, and fruiting bodies in *F. luteovirens* [40]. Soil bacteria and fungi play different roles in affecting soil nutrients, soil enzyme activity, and microbial activity [42]. At present, numerous comparative studies have already been conducted on soil microbial species in the vicinity of the *F. luteovirens* fairy ring. Further research could focus on the effects of enzymes secreted by fungi, particularly those originating from *F. luteovirens*, on the rhizosphere soil environment of plants.

## 4. Biological Characteristics of *F. luteovirens*

### 4.1. Morphological Characteristics of F. luteovirens

**Figure 2:**
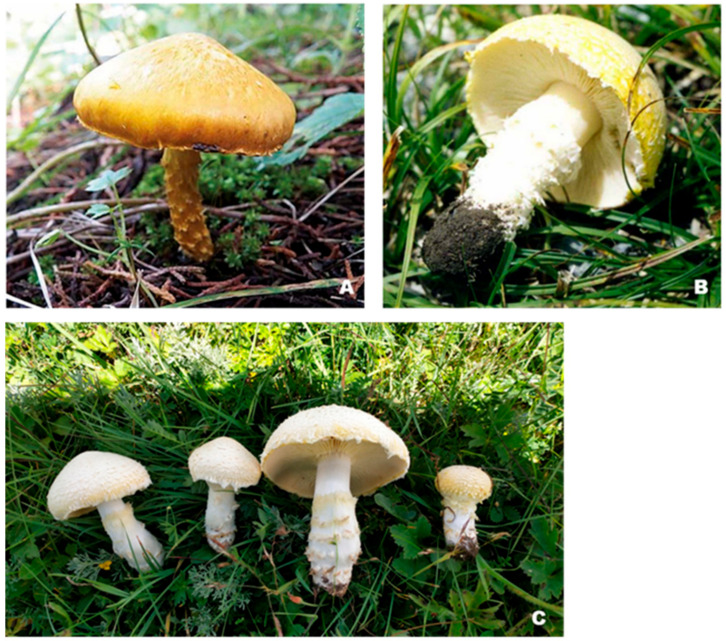
The fruiting bodies of *F. luteovirens* are in different regions. (**A**) reported from Mexico (Arana-Gabriel 2020 [16]); (**B**) reported from the Altai Republic of Russia (Gorbunova 2017 [15]); (**C**) photographed in China (by the author).

The fruiting bodies of *F. luteovirens* are moderately sized and lemon-yellow to sulfur-colored when fresh; when dried, they become practically white (Figure 2). The flesh of the fruiting bodies is thick, white to pale yellow, does not change color after damage, and has a peculiar mushroom odor. The pileus surface is dry and initially spherical but expands after maturity. It is covered with distinct, nearly concentrically arranged scales and measures 5.5–13 cm in diameter. The lamella of the fructification has sparse, yellow, and straight growth towards the pileus, with some curved growth as well. The stipe of the fructification is cylindrical, measuring 2–8 cm in length and 2–2.5 cm in diameter. It has a white to yellow color and is often covered with scales. The interior of the stipe is solid, and the base is frequently swollen. At the base of the stipe, there are spiral arrangements of curled hairs, along with remnants of the partial veil [6,7,43]. However, it is worth noting that there are significant differences in fruiting body morphology between *F. luteovirens* reported from Mexico and *F. luteovirens* reported from China and the Altai Republic of Russia. The population branches of *F. luteovirens* may have undergone different directions of evolution.

**Figure 3:**
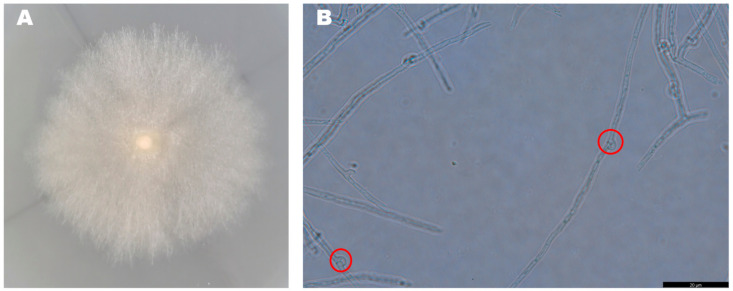
Morphology of the *F. luteovirens* mycelia. (**A**) Colony of isolated *F. luteovirens*; (**B**) Morphology of *F. luteovirens* mycelia. Clamp-connections are indicated by red circles (Photograph by the author).

**Figure 4:**
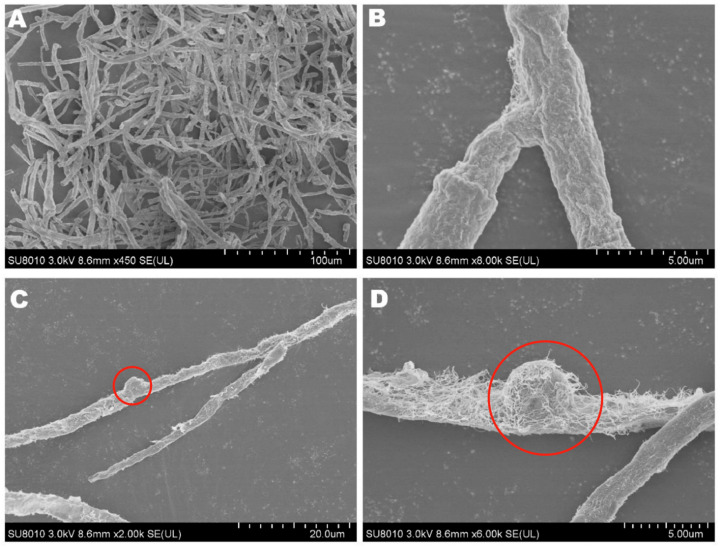
The mycelial structures of *F. luteovirens* were observed with scanning electron microscopy. (**A**) Observation at 100 μm; (**B**,**D**) Observation at 5 μm; (**C**) Observation at 20 μm. Clamp connections are indicated by red circles (Liu 2020 [44]).

The mycelia of the strain isolated from *F. luteovirens* fruiting bodies grew slowly in PDA medium, and the mycelial color gradually transitioned to a yellowish-brown shade in the later stage, eventually ceasing further growth (Figure 3A). Under the light microscope, the structure of the clamp-connection can be distinctly seen (Figure 3B); this observation suggests that the mycelium of *F. luteovirens* is dikaryotic. Scanning electron microscopy showed that the surface of the hypha was smooth, with a thickness of 2–5 μm. The hypha grew straight without any curvature, and mycelia branches could be seen (Figure 4A,B). On the young mycelia, a clamp-connection structure was observed, and the diameter of the arc is approximately 2.5 μm (Figure 4C,D) [44]. 

**Figure 5:**
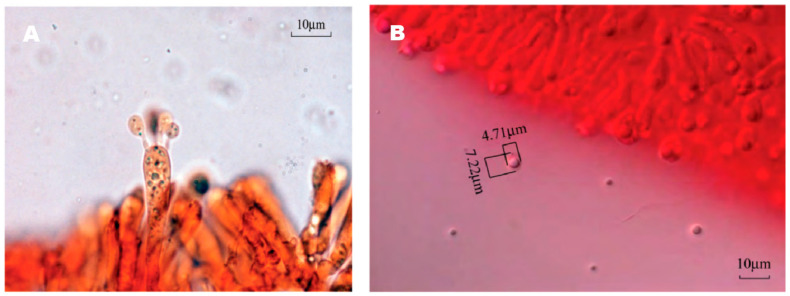
Basidiomata (**A**) and basidiospores (**B**) of *F. luteovirens* (×1000) (Xie 2016 [7]).

**Figure 6:**
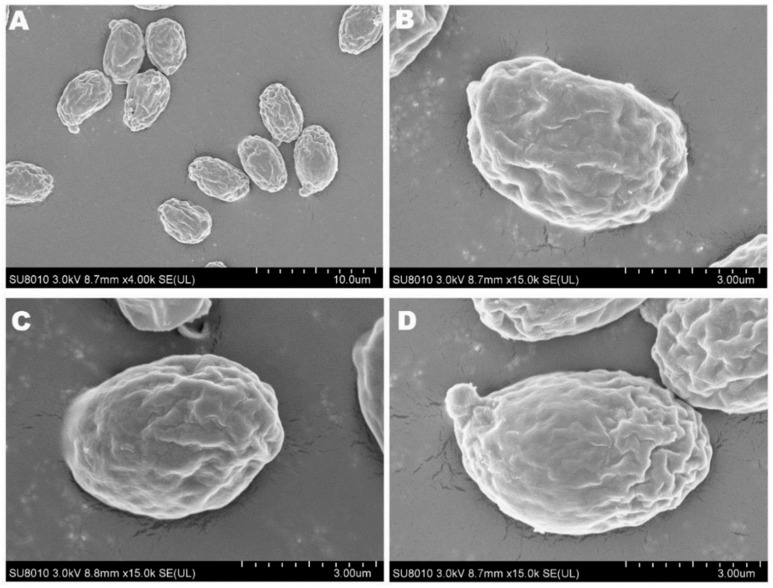
The spores of *F. luteovirens* were observed with scanning electron microscopy. (**A**) Observation at 10 um; (**B**–**D**) Observation at 3 μm (Liu 2020 [44]).

**Figure 7:**
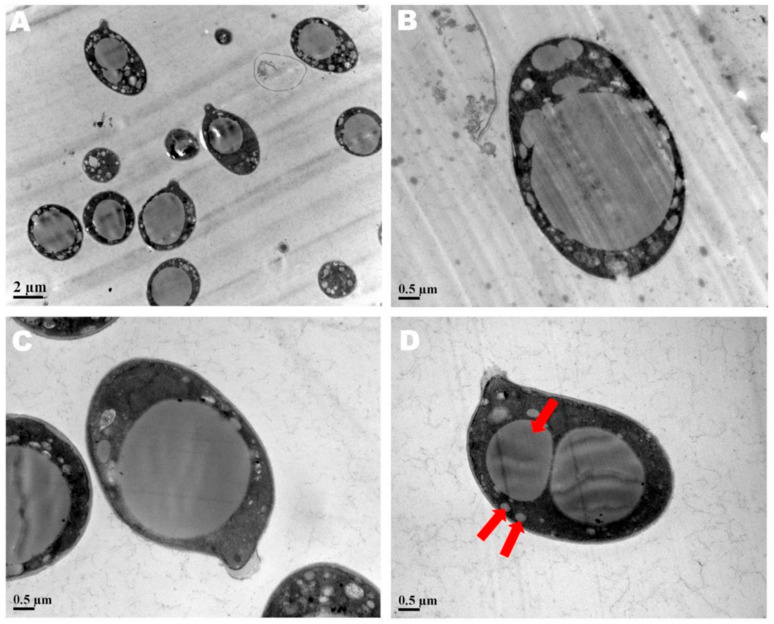
The spores of *F. luteovirens* were observed with a transmission electron microscope. (**A**) Observation at 2 μm; (**B**–**D**) Observation at 0.5 μm. The red arrows indicate vacuoles and lipid droplets within the spore (Liu 2020 [44]).

The spore print of *F. luteovirens* is white. Under the microscope, the basidiomata were rod-shaped, 12.5–16.8 × 4.0–4.8 μm in size, with 4 pedicles and occasionally 2 pedicles. Basidiospores were ellipsoid to spherical, 4.2–8.3 × 3.7–7.5 μm (Q = 1.1–2.2), smooth, and translucent (Figure 5) [7]. Under scanning electron microscopy, the spores of *F. luteovirens* were ellipsoid, with a size of 5.5~6.0 μm × 3.0~3.8 μm (Figure 6). There were some indentations on the surface, and at the base of some spores was an excipuliform appendage (Figure 6D). When the spores of *F. luteovirens* were observed under transmission electron microscopy (Figure 7), it could be seen that there were two layers of spore wall and that the thickness of the spore wall was uneven. Most spores have a vacuole that nearly fills the sporosomal cavity and a few lipid droplets of varying sizes (Figure 7B) [44]. 

### 4.2. Conditions for the Formation of the Fruiting Body

The fruit bodies of *F. luteovirens* are distributed in the grass, either scattered (solitary, scattered, clustered, or clumped) or on the fairy rings. The growth and development process can be divided into five stages: the primary base stage, the bud stage, the growth stage, the umbrella stage, and the decay stage. The growth cycle lasts for 18 to 22 days [43]. This mushroom is ripe from June to September each year, with a harvest period of about 1 to 1.5 months in the same area. The occurrence periods of varying regions differ due to differences in latitude and altitude, with specific times primarily dependent on ground temperature and humidity [17]. The hyphae of *F. luteovirens* could tolerate temperatures ranging from −17.4 °C to −3.8 °C, which is the average temperature of the coldest month. Both temperature and light intensity have an impact on the growth of *F. luteovirens* fruiting bodies throughout their entire growth period. The timing and amount of rainfall directly influence the timing and quantity of mushrooms. The more rainfall there is, the more abundant and robust the mushrooms are. Additionally, precipitation can promote the various growth stages of *F. luteovirens* [6]. According to Xie et al. [7], the fruiting bodies of *F. luteovirens* appear during the summer and autumn seasons, with an average relative humidity of 41~74%, an average temperature of 6.2~15.9 °C, an average annual evaporation of 1393.8~2441.4 mm, an annual precipitation of 344~574 mm, and a humidity coefficient of 0.42~0.78. The Tibetan Plateau is one of the regions with the highest ultraviolet radiation intensity in the world. To cope with this intense UV radiation, *F. luteovirens* produces riboflavin, which results in the mushrooms appearing brighter in high-UV environments [45].

### 4.3. Nutritional Metabolism and Reproductive Mode of F. luteovirens

As a mycorrhizal fungus, *F. luteovirens* does not rely on its own decomposition to obtain nutrients. Studies have shown that the genes related to lignin and cellulose degradation in this fungus are significantly fewer compared to white-rot fungi and brown-rot fungi [13,46,47]. The nutrient requirements of mycorrhizal fungi vary greatly among different species. Most ectomycorrhizal fungi utilize ammonium salts as a source of inorganic nitrogen, while a few can also use nitrates [48]. Current research suggests that *F. luteovirens* is capable of utilizing both ammonium salts and nitrates [49]. KEGG pathway analysis was conducted on transcripts from four major developmental stages of *F. luteovirens*: hyphal stage, primordium stage, immature fruiting body stage, and mature fruiting body stage. This study found significant differential expression of genes involved in cell cycle (ko04111), ribosome (ko03010), MAPK signaling (ko04011), and primary carbohydrate metabolism (ko00010) pathways during reproductive growth [13]. However, it should be noted that this study did not verify the key genes that influence the growth of *F. luteovirens*. In the fruiting body stage, *F. luteovirens* exhibits a complex range of nutrient types. A study utilizing transcriptome sequencing has identified a total of nine significantly different genes associated with carbohydrates. The expression of these genes leads to the breakdown of substances in the soil habitat into monosaccharides and glucose, ultimately completing energy metabolism through the glycolysis pathway and the tricarboxylic acid cycle [50]. At present, the nutritional types of *F. luteovirens* and their symbiotic relationship with plants remain incompletely understood. Further research is necessary to elucidate the adaptation mechanism between *F. luteovirens* and both biological and abiotic factors. Some scholars have studied the small-scale genetic diversity and gene exchange frequency of *F. luteovirens*, revealing that *F. luteovirens* exhibits a relatively large genome. Additionally, only a limited number of new genes were identified during the three-year study, indicating that this species heavily relies on vegetative growth and can persist underground as mycelia for extended periods [51]. The sexual reproductive mating types of edible fungi can be classified into homothallism and heterothallism [52]. However, there is currently no research available on the mating types of *F. luteovirens*. Accurately identifying mating types and genotypes is crucial for artificial domestication cultivation and hybridization breeding.

## 5. Current Status of Artificial Cultivation of *F. luteovirens*

### 5.1. Research on Mycelial Growth

**Figure 8:**
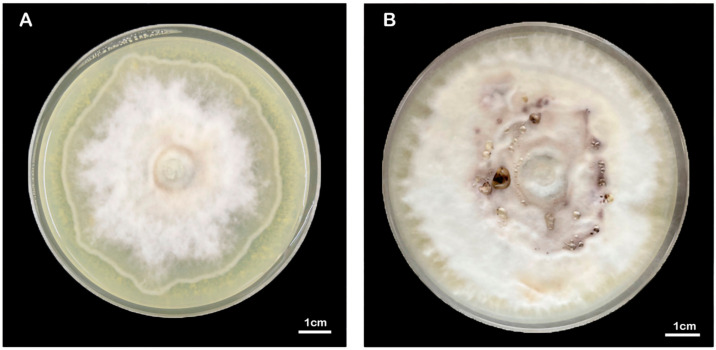
Morphology of the *F. luteovirens* mycelia at different culture days. (**A**) Approximately 40 days, (**B**) 100 days.

Due to the lack of host plants and insufficient exploration of the necessary nutrient elements for the growth of *F. luteovirens* mycelia, the mycelia are unable to obtain enough nutrients under laboratory culture conditions, resulting in a slow growth rate of artificially cultured mycelia. *F. luteovirens* hyphae have a slow growth period from germination to 42 days and then only a fast growth period of about 5 days, which is one of the reasons for the slow growth of the strain. During the stagnant growth period, the hyphae change from their initial white color to a yellowish-brown color, although the mechanism behind this phenomenon is still unclear [53]. The growth temperature range of wild *F. luteovirens* hyphae is 15 °C to 30 °C, the optimum temperature range is 20 °C to 25 °C, and the suitable pH range is 5.5 to 6.5 [54]. Previous experiments have investigated the effects of different carbon and nitrogen sources, traditional Chinese herbal medicine, and growth regulators on the growth of *F. luteovirens* mycelia. Sucrose and peptone are considered more suitable carbon and nitrogen sources, with an optimal carbon-to-nitrogen ratio of 10:1. The most effective mineral nutrient for mycelial growth is MgSO_4_. Licorice and wolfberry extracts, triacontanol, inositol, and selenium have all been found to significantly enhance mycelial growth [55,56,57,58]. Other studies have shown that adding more plant species to the enriched medium improves mycelia growth, possibly due to the fact that mycelia growth depends on certain substances in the plant [50]. Arana-Gabriel et al. [16] conducted a culture screening test on a *F. luteovirens* strain isolated in Mexico and found that the strain reached a growth rate of 0.406 mm/d in a MEA (Malt Extract Agar) medium with a pH of 4. Although this growth rate was higher than previous reports, it was still slower compared to the growth rate of other cultivated or wild fungi. SHI et al. [49] conducted a single-factor experiment and an orthogonal experiment to identify an optimal culture condition. The culture medium consisted of 20% potato extract, 10% soybean sprout extract, 0.3% KH_2_PO_4_, and 1 mg/L VB1 + VB2. After 15 days, the mycelia dry weight could reach 6.5 mg/mL. Some studies have conducted statistical analyses on the number of clamp connections in *F. luteovirens* strains with excellent, good, and medium hyphae growth rates, and combined analysis indicates that *F. luteovirens* exhibits a faster hyphae growth rate and a greater average dry weight when there are fewer clamp connections [59]. Additionally, many fungi possess the ability to bioenrich selenium. Studies have shown that selenium can enhance the growth of *F. luteovirens* hyphae and significantly increase its biomass. The optimal concentration for selenium-enriched cultures is found to be 0.80 mmol/L [60]. To date, none of the aforementioned studies have successfully identified a comprehensive formula that is highly conducive to the growth of *F. luteovirens* mycelium. We are currently conducting research in this field and have successfully achieved dense growth of *F. luteovirens* mycelium on the composite medium (Figure 8). If we make more discoveries and progress, we will share the detailed results of this experiment with the public.

### 5.2. Preliminary Study on Artificial Cultivation

In recent years, numerous researchers have attempted to domesticate *F. luteovirens*; however, no successful cases have been reported thus far. The growth of this particular type of mycorrhizal fungus necessitates a specialized culture medium and specific environmental conditions, including complex formulations, specific soil compositions, and nutrient requirements. In natural settings, mycorrhizal fungi rely on plant roots to obtain essential nutrient elements, transitioning from the vegetative stage to the reproductive stage and ultimately forming fruiting bodies [61]. This reliance on plant roots poses a challenge for artificial cultivation. It has previously been reported that a Japanese scholar processed the mycelia of *F. luteovirens* with trace nitrite ions, and the granular mycelia produced was incubated at a low temperature below 12 °C. When urea was added to the medium, a flat mushroom-like mycelium with a diameter of about 7 cm could be obtained in about 1 month [62]. LIU conducted the same experimental method as mentioned above. After adding potassium nitrite to the culture for a period of 6 months, the shaker bottle was filled with *F. luteovirens* mycelium, but no *F. luteovirens* fruiting bodies were observed [44]. We speculate that the success of the previous experiment was based on chance, or it could be attributed to inherent differences in the strains that led to the non-replicability of the experiments. WEI obtained the test strain by isolating the fruiting body of wild *F. luteovirens* [54]. In seed mycelium, it was observed that the pollution rate of the grain medium was high. The absorption and utilization of cottonseed shell medium were not ideal, while grass powder medium exhibited a relatively good growth rate in the later period. After soil-covering culture, the hyphae on the grass powder medium were able to form the primordia and yellow fruiting bodies. However, when the diameter of the pileus was about 1 cm, the fruiting bodies would appear to undergo autolysis and death. Insufficient temperature, light, water, and nutrients are all possible factors that adversely affect the normal growth of the hyphae. Currently, the key conditions for artificial cultivation of *F. luteovirens* have not been identified. The imitation of wild cultivation of *F. luteovirens* has been proposed [63]; however, the culture of the mycelium growth stage has not been successful yet, so this artificial domestication technology has not been attempted. Furthermore, there are limited reports on the cultivation of *F. luteovirens*, and no significant progress has been made in understanding its primordium and fruiting body formation.

## 6. Nutritional Components of *F. luteovirens*

### 6.1. Basic Components

The dry fruiting body of *F. luteovirens* contains 85.92% dry matter, 46% crude protein, 3.85% crude fat, 6.46% crude fiber, 18.63% soluble sugar, and 10.98% ash. The fruiting bodies contain 18 kinds of amino acids (including eight kinds of essential amino acids), vitamin B1, vitamin B2, vitamin C, and vitamin E, as well as P, K, Fe, Mg, Zn, and other minerals essential to the human body [6,64]. Li et al. compared the nutritional components of *F. luteovirens* with *Lentinula edodes* (*L. edodes*) and *Agaricus bisporus* (*A. bisporus*) (Table 1 and Table 2) and found that *F. luteovirens* had significantly higher protein and ash contents. It is a precious wild edible mushroom with a high protein and mineral content [64].

### 6.2. Polysaccharide

The polysaccharide content in *F. luteovirens* is comparable to that in lentinus mushrooms, reaching 3.56~8.12 g/100 g [65]. Additionally, the polysaccharide content in mycelium water extract is approximately 31.21% [66]. Analysis reveals that the polysaccharides of *F. luteovirens* primarily consist of xylose and Arabic sugar [67], and they predominantly contain six sugar residues: (A)→4)-β-D-Manp-(1→; (B)→3)-α-L-Fucp-(1→; (C) α-L-Araf-(1→; (D)→6)-β-D-Galp-(1→; (E)→4)-α-D-GlcAp-(1→; (F)→3)-β-D-Glcp-(1→ [44].

### 6.3. Volatile Components

Analysis of the volatile fat-soluble components of *F. luteovirens* showed that the fruiting body was rich in fatty acids, esters, alkenes, and other compounds, with the highest relative content being linoleic acid (ω-6, 48.2%) [68]. Based on GC–MS analysis, a total of 25 fatty acid components and contents in the fruity bodies of *F. luteovirens* were identified, among which the relative contents of polyunsaturated fatty acid accounted for 10.6%, monounsaturated fatty acid accounted for 31.5%, and saturated fatty acid accounted for 56.9% [69]. Genomic analysis of *F. luteovirens* showed that there were 672 genes related to cell metabolism of terpenoids and polyketones, accounting for a large proportion, indicating that *F. luteovirens* is a potential resource species of terpenoids and polyketones [13].

### 6.4. Others

The selenium content in the fruiting body of *F. luteovirens* ranges from 0.57 to 2.53 mg/kg, which meets the standard for selenium-rich food. Additionally, various nucleosides (such as uridine, deoxyuridine, uracil, guanosine, inosine, and adenosine), esters, and sterols were isolated from the fruiting body [70,71].

## 7. Medicinal Value of *F. luteovirens*

*F. luteovirens* mushrooms are a traditional Tibetan herbal medicine. Historical records indicate that it has a sweet taste and provides benefits to the stomach and intestines. Regular consumption of these mushrooms could reduce blood cholesterol levels. Moreover, it is also used to alleviate symptoms such as dizziness, headache, neurasthenia, and insomnia and has been found to have preventive effects against viral infections and diseases like beriberi. It has a high content of iron and phosphorus and is also an ideal health food for benefiting qi and nourishing blood. Due to its rich nutritional value, it also has pharmacological effects on promoting children’s development and enhancing humans’ immunity [6,72,73].

### 7.1. Antioxidant and Anti-Aging Ability

Polysaccharides are significant components of edible fungi with both nutritional and medicinal value. Research has shown [74,75,76,77,78] that the scavenging ability of *F. luteovirens* polysaccharides towards DPPH is superior to that of *Lentinula edodes* polysaccharides, and the scavenging ability towards ABTS is better than that of *Hericium erinaceus* polysaccharides. Moreover, it has a higher moisture absorption rate than *Cordyceps sinensis* polysaccharides and a higher moisture retention rate than *Sargassum horneri* polysaccharides. *F. luteovirens* polysaccharides after extraction and separation can effectively increase the activities of SOD, GSH-Px, and CAT in PC12 cells while decreasing the production of ROS and MDA, thus protecting PC12 cells from H_2_O_2_-induced oxidative stress [79]. The protoilludane sesquiterpene aryl esters extracted from the fruiting bodies of *F. luteovirens* can have good scavenging efficiency on DPPH free radicals [80]. A highly polar free radical inhibitor, L-(+)-Ergothioneine [81], has been successfully extracted from the methanolic extract of *F. luteovirens*. It is a natural antioxidant that offers significant cell protection and is non-toxic. Additionally, it has the ability to chelate heavy metal ions and safeguard red blood cells against free radical damage.

### 7.2. Anti-Tumor and Anticancer Potential

The exopolysaccharides of *F. luteovirens* have been found to inhibit tumor cell proliferation while having no side effects on normal cells [79]. Polysaccharides play a role in enhancing the immune function of the body by regulating humoral immunity and regulating the expression of immune-related genes [82,83]. This leads to the promotion of the proliferation and activity of immune cells, ultimately resulting in an anti-tumor effect. The small-molecule isolates from *F. luteovirens* exhibited potent antitumor activity by inducing apoptosis of lung cancer cells through the activation of caspase-3. This isolate consisted of six amino acids, two nucleosides, two glycosides, two terpenoids, one phenylpropanoid compound, one ester, and one alkaloid [84]. Previous studies have demonstrated that a lectin called ALL [85], which was isolated from *F. luteovirens*, has the ability to inhibit the proliferation of tumor cell lines L1210 (leukemia), MBL2 (leukemia), and HeLa (cervical). Furthermore, this lectin exhibited greater stability compared to typical mushroom lectins and plant lectins, as it retained its activity even at a temperature of 70 °C.

### 7.3. Anti-Diabetic Activity

The water extract of *F. luteovirens* has certain benefits in the treatment of diabetes; it can significantly improve the oral glucose tolerance of rats with type 2 diabetes, significantly increase the level of SOD in the serum of rats, and reduce the expression of MDA and four inflammatory factors [86]. Additionally, the polysaccharides in it can effectively improve and alleviate renal tissue damage caused by hyperglycemia [87].

### 7.4. Other Functions

The water extract of *F. luteovirens* also demonstrates a significant anti-inflammatory and analgesic effect on experimental migraine rats. This extract effectively reduces vasodilation and alleviates stimulation of the trigeminal nerve [88]. These findings provide a plausible explanation for the historical use of *F. luteovirens* mushrooms in ancient medical books to treat headaches.

## 8. Future Prospects

### 8.1. Strengthen Resource Protection

*F. luteovirens*, as an edible mushroom, is known for its delicious taste and rich nutrition. It has become increasingly popular among people, with the market price for fresh mushrooms reaching as high as six or seven hundred yuan per kilogram during the harvest season. Higher-quality mushrooms can even be sold for thousands of yuan. However, in their pursuit of economic benefits, local residents have been expanding the number and area of pickings, resulting in a constant reduction in the number of fruiting bodies [7]. Excessive picking can have detrimental effects on the grassland ecological system. To ensure the long-term survival of *F. luteovirens,* it is important to promote the concept of sustainable development among local residents. In cases where it is necessary, certain protected areas can be established to safeguard the population of *F. luteovirens*. While the majority of resources for researching the *F. luteovirens* species are currently derived from Qinghai and Tibet, it is important not to disregard the potential resources available in other regions such as Sichuan and Gansu. It may be necessary to establish *F. luteovirens* species banks in research institutes or universities to ensure the long-term preservation of essential germplasm resources and their genetic information. This would provide a foundation for future genetic breeding and artificial domestication research.

### 8.2. Promote the Process of Artificial Domestication

*F. luteovirens*, a rare edible mushroom resource in the Qinghai–Tibet Plateau, thrives in a distinctive growing environment and forms mycorrhizal symbiosis with *K. humilis*; artificial cultivation of this mushroom has not yet been achieved. The challenge of artificially domesticating edible mycorrhizal fungi lies in their separation from the symbiotic host plant. When placed under pure culture conditions, these fungi often exhibit slow or stunted growth, emphasizing the importance of the symbiotic system in addressing this issue. There have been successful cases in which *Pinus armandii Franch* seedlings were inoculated with spore suspensions of *Tuber melanosporum Vittad* for the synthesis of ectomycorrhizal seedings [89]. Mycorrhizal synthesis of *Tricholoma matsutake* and pine seedlings can harvest fruiting bodies in field experiments. Although the colonized *T. matsutake* may be replaced by the original dominant ECM fungi during the growth of pines [90], it is still feasible to artificially construct an efficient symbiotic system of mycorrhizal edible fungi. The artificial domestication study of *F. luteovirens* can also try to infect the roots of *K. humilis* with *F. luteovirens* to observe the potential formation of mycorrhizal seedlings. Another approach is to simulate wild cultivation by artificially sowing the cultivation medium covered with hyphae in the growth areas of *K. humilis* and other forages to observe the occurrence of hyphae. In China, mycorrhizal edible fungi account for approximately 70% of the total number of edible fungi [11]. Breaking through the challenges of artificially cultivating mycorrhizal edible fungi would not only advance cultivation technology but also have significant economic and practical value.

### 8.3. Development and Utilization of Edible and Medicinal Value

*F. luteovirens* has high food and medicinal value. In addition to supplementing nutrition and improving diet balance, it also has the effect of enhancing immunity, making it an ideal raw material for developing health foods. Currently, *F. luteovirens* fruiting bodies are mostly sold in dried form, and there are few processed products available. By processing the mushrooms into various flavored foods, consumers can have a wider range of choices. Since this mushroom is a local specialty and not well-known nationwide, effective brand building and commercial publicity are necessary to enhance its economic value. *F. luteovirens* contains a variety of active ingredients, particularly those with antioxidant and anti-tumor properties. If the active ingredients can be isolated and purified, it is expected to lead to the development of new cancer treatment drugs. The advancement of omics technology, including genomics and metabolomics, allows for a more comprehensive and systematic study of these active components. Some researchers have explored the extraction of biosynthetic genes and gene clusters from slow-growing basidiomycetes and transferring them to faster-growing host organisms [91]. This approach provides a new avenue for studying the diversity of metabolites in *F. luteovirens* and further advancing their development and utilization. Many mushroom strains can be grown in submerged liquid to produce a variety of bioactive substances such as proteins, carbohydrates, enzymes, lipids, etc. These substances find applications in the food industry and drug production [92]. Compared to direct culture of fruiting bodies, this controlled bioreactor culture method is more convenient and efficient. Although it is currently not feasible to artificially cultivate *F. luteovirens* for mushroom production, a promising approach is to concentrate on the submerged culture of *F. luteovirens* mycelium for functional components.

## 9. Conclusions

*F. luteovirens* is a rare edible mushroom resource growing at an altitude of 3000 m above sea level and forms a symbiotic relationship with *K. humilis*. It has a high nutritional value, provides a variety of essential nutrients for the human body, and can also regulate the body’s immune ability. Existing studies have demonstrated the antioxidant, anti-aging, and anti-tumor activity of *F. luteovirens*, as well as its potential in the treatment of diabetes and migraine. *F. luteovirens* cannot currently be artificially cultivated; thus, we must first safeguard this species to prevent overharvesting and harm to the biological habitat of alpine meadows or grasslands. At the same time, we still require more researchers to thoroughly examine the interaction between *F. luteovirens* and the grassland ecosystem, solve the puzzle of the fruiting body’s formation mechanism, and investigate the functional component in it in order to create new beneficial health products or medications.

## Figures and Tables

**Table 1:** Comparison of physicochemical properties and chemical constituents of the dry fruiting bodies of *F. luteovirens*, *L. dodes*, and *A. bisporus*.

Species Tested	Main Nutritive Components Content/%				
	Crude Protein	Crude Fat	Crude Fiber	Soluble Sugar	Ash
*F. luteovirens*	46.00	3.85	6.46	18.63	10.98
*L. edodes*	27.16	2.24	8.14	17.68	5.93
*A. bisporus*	35.89	2.05	6.10	22.64	7.83
**Species Tested**	**Vitamins Content/[mg × (100 g)^−1^]**				
	**VA**	**VC**	**VB1**	**VB2**	**VE**
*F. luteovirens*	-	9.78	1.53	9.28	3.37
*L. edodes*	-	3.35	0.07	1.13	0.79
*A. bisporus*	-	5.54	0.10	1.10	5.38
**Species Tested**	**Mineral Elements Content/(g × Kg^−1^)**				
	**P**	**K**	**Fe**	**Mg**	**Zn**
*F. luteovirens*	12.76	30.24	0.39	0.87	0.08
*L. edodes*	10.01	20.35	0.07	1.07	0.08
*A. bisporus*	10.11	20.54	0.32	0.66	0.05

**Table 2:** Comparison of amino acid content in the fruiting bodies of *F. luteovirens*, *L. dodes*, and *A. bisporus*.

Amino Acids Components	Amino Acids Content/[g × (100 g)^−1^]		
	*F. luteovirens*	*L. edodes*	*A. bisporus*
Aspartic acid (ASP)	2.24	1.61	2.01
Threonine (THR) *	1.31	0.99	1.10
Serine (SER)	1.57	1.02	1.10
Glutamic acid (GLU)	6.98	4.83	4.61
Proline (PRO)	0.73	0.54	1.29
Glycine (GLY)	1.83	0.93	1.05
Alanine (ALA)	1.95	1.08	1.95
Cystine (CYS)	0.31	0.23	0.20
Valine (VAL) *	0.77	0.70	0.84
Methionine (MET) *	1.22	0.62	1.00
Isoleucine (ILE) *	0.90	0.70	0.88
Leucine (LEU) *	1.64	1.29	1.64
Tyrosine (TYR)	0.62	0.44	0.46
Phenylalanine (PHE) *	0.98	0.89	0.97
Histidine (HIS)	0.83	0.40	0.47
Lysine (LYS) *	1.20	0.95	1.08
Arginine (ARG)	1.30	0.93	1.01
Tryptophan (TRP) *	-	-	-
Total amino acids (TAA)	26.38	18.15	21.66
Essential amino acids (EAA)	8.02	6.14	7.51

Note: * stands for the essential amino acid (EAA).

## Data Availability

Data sharing is not applicable to this article, as no new data was created or analyzed in this study.
